# ANO1/TMEM16A interacts with EGFR and correlates with sensitivity to EGFR-targeting therapy in head and neck cancer

**DOI:** 10.18632/oncotarget.3277

**Published:** 2015-03-16

**Authors:** Anke Bill, Abraham Gutierrez, Sucheta Kulkarni, Carolyn Kemp, Debora Bonenfant, Hans Voshol, Umamaheswar Duvvuri, L. Alex Gaither

**Affiliations:** ^1^ Novartis Institutes for Biomedical Research, Cambridge, MA 02139, USA; ^2^ University of Pittsburgh, Medical Center, Department of Otolaryngology, Pittsburgh, PA 15213, USA; ^3^ Novartis Institutes for Biomedical Research, Basel, CH-4002, Switzerland; ^4^ VA Pittsburgh HealthCare System, Pittsburgh, PA 15213, USA

**Keywords:** epidermal growth factor receptor (EGFR), EGFR-targeted therapy, biomarker, calcium-activated chloride channel, protein-protein interaction

## Abstract

The epidermal growth factor receptor (EGFR) contributes to the pathogenesis of head&neck squamous cell carcinoma (HNSCC). However, only a subset of HNSCC patients benefit from anti-EGFR targeted therapy. By performing an unbiased proteomics screen, we found that the calcium-activated chloride channel ANO1 interacts with EGFR and facilitates EGFR-signaling in HNSCC. Using structural mutants of EGFR and ANO1 we identified the trans/juxtamembrane domain of EGFR to be critical for the interaction with ANO1. Our results show that ANO1 and EGFR form a functional complex that jointly regulates HNSCC cell proliferation. Expression of ANO1 affected EGFR stability, while EGFR-signaling elevated ANO1 protein levels, establishing a functional and regulatory link between ANO1 and EGFR. Co-inhibition of EGFR and ANO1 had an additive effect on HNSCC cell proliferation, suggesting that co-targeting of ANO1 and EGFR could enhance the clinical potential of EGFR-targeted therapy in HNSCC and might circumvent the development of resistance to single agent therapy. HNSCC cell lines with amplification and high expression of ANO1 showed enhanced sensitivity to Gefitinib, suggesting ANO1 overexpression as a predictive marker for the response to EGFR-targeting agents in HNSCC therapy. Taken together, our results introduce ANO1 as a promising target and/or biomarker for EGFR-directed therapy in HNSCC.

## INTRODUCTION

Head and neck squamous cell carcinoma (HNSCC) is the sixth most common cancer worldwide, with a predicted 40,000 new cases and 8,000 deaths in the USA in 2014 [1]. The current standard of care involves a multistrategic therapy with surgery, radiation therapy and chemotherapy. Despite advances in therapy, long-term survival hasn’t improved significantly over the last decade, with half of the patients succumbing within 1 to 5 years [1–3].

The epidermal growth factor receptor (EGFR) is overexpressed in approximately 90% of HNSCC and has been implicated in the pathogenesis of head and neck cancers. EGFR (ErbB1) is a transmembrane tyrosine kinase receptor which, together with HER2/ErbB2, HER3/ErbB3 and HER4/ErbB4, composes the family of HER/ErbB proteins [4, 5]. Activation of EGFR by specific ligands including EGF, TGF-alpha, HB-EGF and amphiregulin induces the homo- and hetero-dimerization of EGFR with other ErbB proteins and leads to the phosphorylation of tyrosine residues in the cytoplasmic domain of the receptor. These phosphotyrosine residues then serve as docking sites for proteins which initiate the activation of key signaling pathways like the mitogen-activated protein kinase (MAPK)- or the PI3K-activated protein kinase B (AKT)-pathway; key regulators of HNSCC cell proliferation, tumor growth, invasion and metastasis [4, 6].

Overexpression of EGFR and EGFR gene amplification have been correlated with decreased survival of patients with HNSCC [3, 7, 8]. Inhibition of EGFR using a variety of therapeutic approaches diminishes tumor growth in preclinical HNSCC models [9, 10]. However, despite the ubiquitous expression of EGFR in HNSCC tumors, only a subset of patients responds to EGFR-directed therapy in clinical trials [3]. Accordingly, small-molecule tyrosine kinase inhibitors (e.g. Gefitinib) and monoclonal antibodies against EGFR (e.g. Cetuximab) have shown only limited efficacy in HNSCC patients when used as monotherapy [11–13]. EGFR expression or amplification does not consistently predict response to EGFR targeted therapies [14]. Mutations in the tyrosine kinase domain of EGFR that are known to associate with response or resistance to EGFR therapy in other cancers are rare in patients with HNSCC and do not correlate with efficacy of EGFR-inhibition or clinical outcome [15]. Further research is needed to elucidate the molecular mechanisms underlying the response to EGFR-inhibition in HNSCC and to characterize biomarkers which would allow for the identification of individuals who are likely to benefit from EGFR-targeting strategies as well as for the development of more effective mono- and combinatorial therapeutic approaches for the treatment of HNSCC.

ANO1 (TAOS2, DOG1, ORAOV2, TMEM16A) is a calcium-activated chloride channel [16–18] expressed on the plasma membrane of secretory epithelia, smooth muscles and sensory neurons [19–24]. ANO1 mediates transepithelial ion transport and exhibits an important function in regulating airway fluid secretion, gut motility, secretory functions of exocrine glands, renal function, (vascular) smooth muscle contraction and nociception [22, 24, 25]. Dysfunction of ANO1 is associated with several disease states including cystic fibrosis, asthma, gastroparesis, hypertension, rota-virus induced diarrhea and polycystic kidney disease [26–30].

ANO1 is amplified and highly expressed in a large subset of HNSCC tumors, as well as in a variety of other carcinomas including breast cancer, prostate carcinoma, glioblastoma, GIST (gastrointestinal stromal tumor) and ESCC (esophageal squamous cell carcinoma) [31–37]. The gene encoding for ANO1 maps to a region on chromosome 11 (11q13) that is frequently amplified in HNSCC [38]. ANO1 was originally interpreted as a passenger gene amplification and only recent studies have shed new light on the role of ANO1 in tumorigenesis. Knockdown and small-molecule facilitated degradation of ANO1 impairs HNSCC and ESCC cell proliferation and have been shown to correlate with the inhibition of mitogen-activated kinase (MAPK) and protein kinase B (AKT) signaling [33, 34, 39]. Furthermore, ANO1 expression has been reported to promote tumorigenesis by activating epidermal growth factor receptor (EGFR) and calmodulin dependent kinase (CAMK)-signaling [33]. A recent study has identified ANO1 as a switch between the proliferative and metastatic phenotype of human HNSCC cells by affecting the transition of cells from the epithelial to a mesenchymal state [40]. High expression of ANO1 in HNSCC, ESCC and prostate cancer correlates with a higher risk of distant metastasis and a shorter survival of these patients [31, 35, 41, 42]. Mechanistically, it has been shown that ANO1 protein levels rather than ANO1-dependent chloride conductance are critical for ANO1-dependent cell proliferation [39], indicating a channel-independent function of ANO1 in promoting cell proliferation, possibly by ANO1 interacting with other proteins on the cell membrane. However, it remains unclear how ANO1 exhibits its function on cellular signaling pathways to promote cancer growth.

Here we investigated the mechanism underlying ANO1′s function as an activator of cellular signaling and promoting tumor growth by performing an unbiased proteomics discovery approach. We identified ANO1 to interact with EGFR in HNSCC cells. Using functional and structural mutants of EGFR and ANO1 we found the trans/juxtamembrane domain of EGFR to be critical for the interaction of EGFR and ANO1, while the C-terminus of ANO1 was not required. The interaction of ANO1 and EGFR was not dependent on the phosphorylation of EGFR or the activity of ANO1. Our results show that ANO1 and EGFR form a functional complex to jointly regulate cell proliferation. Expression of ANO1 affected EGFR stability and expression while EGFR-signaling elevated ANO1 protein levels, establishing a functional and regulatory link between ANO1 and EGFR in HNSCC cells. Co-inhibition of EGFR and ANO1 had an additive effect on head and neck cancer cell proliferation, suggesting that co-targeting of ANO1 and EGFR could enhance the clinical potential of EGFR-targeted therapy in HNSCC. Consistent with ANO1 being a regulator of EGFR-signaling, HNSCC cell lines with amplification and high expression of ANO1 showed enhanced sensitivity to Gefitinib, suggesting ANO1 overexpression as a predictive marker for the response to EGFR-targeting agents in HNSCC therapies. Taken together, our results introduce ANO1 as a promising target and/or biomarker for EGFR-directed therapy in HNSCC.

## RESULTS

### ANO1 and EGFR form a complex in head and neck cancer cells

**Figure 1:**
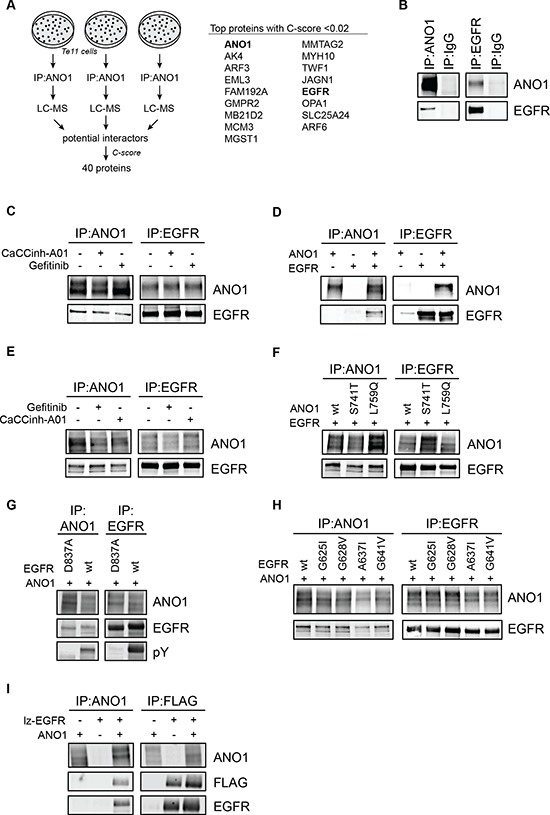
ANO1 and EGFR form a complex in HNSCC cells **(A)** Summary of discovery proteomics experiments after ANO1-pulldown in Te11 cells. ANO1 was immunoprecipitated from Te11 cell lysates and proteins co-purified with ANO1 were analyzed by LC-MS. 40 proteins were identified to interact with ANO1 in all three experiments with a C-score < 0.1. The top proteins with a C-score < 0.02 are shown. The full list of identified proteins is included as Supplementary Table 1. **(B)** Immunoblot after immunoprecipitation of ANO1 (left) or EGFR (right) from Te11 cell lysates using an anti-ANO1 or anti-EGFR antibody coupled to magnetic beads. IgG was used a control. Eluted proteins were run on a western blot and probed with antibodies against ANO1 and EGFR. Representative immunoblots are shown. **(C)** Immunoprecipitation of ANO1 and EGFR in Te11 cell lysates after 24 h treatment with Gefitinib (1 μM) or CaCCinh-A01 (10 μM). **(D)** Immunoprecipitation of ANO1 and EGFR in HEK293T cell lysates. HEK293T cells were transfected with equal amounts of plasmids encoding EGFR, ANO1 or both plasmids and EGFR/ANO1 complexes were analyzed as in Figure 1B. The band for EGFR in the first lane of the IP against EGFR represents endogenous EGFR. **(E)** Immunoprecipitation of ANO1 and EGFR in HEK293T cell lysates after 24 h treatment with Gefitinib (1 μM) or CaCCinh-A01 (10 μM). **(F)** Immunoprecipitation of ANO1-mutants and EGFR in HEK293T cell lysates. HEK293T cells were transfected with equal amounts of plasmids encoding EGFR and ANO1-wt, -S741T (constitutively active) or -L759Q (inactive). **(G)** Immunoprecipitation of ANO1 and an EGFR-kinase mutant in HEK293T cell lysates. HEK293T cells were transfected with equal amounts of plasmids encoding EGFR-wt or an inactive kinase mutant (EGFR-D837A) and ANO1. **(H)** Immunoprecipitation of ANO1 and EGFR-dimerization mutants in HEK293T cell lysates. HEK293T cells were transfected with equal amounts of plasmids encoding ANO1 and EGFR-wt or dimerization mutants of EGFR. **(I)** Immunoprecipitation of ANO1 and FLAG-lz-EGFR in HEK293T cell lysates. HEK293T cells were transfected with equal amounts of plasmids encoding ANO1, lz-EGFR or both plasmids. ANO1 /lz-EGFR complexes were analyzed by immunoprecipitation using an anti-ANO1 or anti-FLAG antibody coupled to magnetic beads and by immunoblotting of the eluted proteins.

One hypothesis for the mechanism underlying ANO1′s channel-independent function in promoting EGFR-signaling and cell proliferation is that ANO1 exhibits its function in cancer cells by interacting with membrane proteins. To test this hypothesis, we characterized the protein interactome of ANO1 under endogenous expression in the HNSCC cell line Te11 using a discovery proteomics approach. Te11 cells express high levels of ANO1 and loss of ANO1 expression by RNAi or compound-mediated degradation has been shown to inhibit Te11 cell proliferation and to reduce EGFR-signaling [33, 39]. Te11 cells were lysed and ANO1 protein complexes were captured with an ANO1-specific antibody coupled to magnetic beads. The precipitated ANO1-protein complexes were purified by PAGE and analyzed by LC-MS. Proteins found to bind unspecifically to the beads in the absence of ANO1-antibody were removed. The experiment was repeated independently three times and only proteins identified in all three experiments with two or more unique peptides were considered. In order to filter for false-positive interactors and to identify the most significant protein interactions we rank ordered the list of identified proteins according to the frequency of unspecific interaction in the CRAPome repository (see Method section for more details). Based on these criteria, a total of 40 proteins were identified as potential interactors of ANO1. EGFR was found to be the highest ranking plasma membrane protein besides ANO1 among the proteins with the lowest C-score (Figure 1A and Supplementary Table 1). Since ANO1 has been shown to regulate EGFR-signaling in Te11 cells, we set out to further explore the observed interaction between ANO1 and EGFR. To confirm the interaction of ANO1 and EGFR we performed immunoprecipitation of ANO1 in Te11 cell lysates and analyzed proteins co-captured together with ANO1 by western blot. EGFR coimmunoprecipitated when ANO1 was captured with an ANO1-specific antibody and showed no binding when an unspecific antibody was used as a control. The interaction of ANO1 and EGFR was also detectable under reciprocal conditions when an EGFR-specific antibody was used to capture EGFR and co-bound ANO1 (Figure 1B). Furthermore, ANO1 and EGFR showed a significant amount of membrane colocalization in Te11 cells (Supplementary Figure 1C). These findings support the conclusion that ANO1 and EGFR form a complex in Te11 cells. To explore whether the interaction of ANO1 and EGFR was limited to Te11 cells or whether ANO1 and EGFR also interact in other cancer cell lines, we immunoprecipitated EGFR or ANO1 from lysates of OE21 and SCC4 cells, two HNSCC cells lines with amplification and high expression of ANO1, and probed for coimmunoprecipitation of ANO1 and EGFR, respectively, by western blotting (Supplementary Figure 1A). In addition to Te11 cells, we found ANO1 and EGFR to form a complex also in OE21 and SCC4 cells, suggesting a potential functional role of the interaction between ANO1 and EGFR in HNSCC cell lines.

### The interaction between ANO1 and EGFR does not depend on either proteins activity

To explore whether the interaction of EGFR and ANO1 is dependent on the calcium-activated chloride channel (CaCC) function of ANO1 or the kinase activity of EGFR, we treated Te11 cells with the ANO1-inhibitor CaCCinh-A01 or the EGFR-kinase inhibitor Gefitinib (Iressa) before analyzing the interaction of EGFR and ANO1 by immunoprecipitation (Figure 1C). Neither treatment affected the interaction of ANO1 and EGFR in Te11 cells, suggesting that ANO1 and EGFR form a complex independent of the activation state of either protein. Because chemical inhibition of EGFR- or ANO1-activity is transient and compound is not present during the immunoprecipitation process, we investigated the functional requirements for the interaction of ANO1 and EGFR using mutants of ANO1 and EGFR with altered functional properties. For this, we used HEK293T cells, which do not express ANO1 and express low levels of EGFR, as a model system to reconstitute the interaction of EGFR and ANO1. Coexpression of ANO1 and EGFR resulted in significant coimmunoprecipitation of both proteins when an ANO1- or EGFR-specific antibody was used, but no signal was detectable when only one protein was expressed (Figure 1D). Furthermore, ANO1 and EGFR showed significant colocalization when expressed in HEK293T, strengthening the hypothesis that ANO1 and EGFR form a complex (Supplementary Figure 1C). In agreement with the results obtained in Te11 cells, treatment with CaCCinh-A01 or Gefitinib did not affect the interaction of ANO1 and EGFR (Figure 1E). We next tested whether a mutant of ANO1 (ANO1-L759Q) devoid of CaCC-activity but with stable expression on the plasma membrane or a mutant of ANO1 with increased calcium-sensitivity and constitutive CaCC-activity (ANO1-S741T) [43] would interact with EGFR. ANO1-wt, -L759Q or -S741T were transfected together with EGFR into HEK293T cells and ANO1 or EGFR were immunoprecipitated. Consistent with the lack of effect of chemical inhibition of ANO1 by CaCCinh-A01, neither activation of ANO1 by the S741T mutation nor a mutation rendering ANO1 inactive had an effect on the interaction between ANO1 and EGFR and both mutants coimmunoprecipitated at a similar level as ANO1-wt (Figure 1F). Similarly, coexpression of ANO1 with a kinase-dead mutant of EGFR [44] did not alter EGFR's ability to interact with ANO1 (Figure 1G). Next we asked whether the dimerization of EGFR might be necessary for EGFR to interact with ANO1. For this, we coexpressed ANO1 with mutants of EGFR with impaired dimerization [45]. All tested dimerization mutants of EGFR interacted with ANO1 to the same extend as wildtype EGFR, suggesting that the interaction of EGFR and ANO1 is independent of the dimerization status of EGFR (Figure 1H). To obtain further evidence for this hypothesis we analyzed whether ANO1 interacts with constitutively dimerized receptors. A constitutively dimerized EGFR (lz-EGFR) was constructed by replacing the extracellular domain of the receptor with a FLAG-tagged dimerization module consisting of a leucine zipper and a single cysteine residue that forms a disufilde bridge upon dimerization [46]. When coexpressed with ANO1 in Hek293T cells, lz-EGFR interacted with ANO1 at a similar level as the full-length EGFR, indicating that the interaction of ANO1 and EGFR is independent of the dimerization status of EGFR (Figure 1I). Taken together, these findings demonstrate that the interaction between ANO1 and EGFR is independent of ANO1′s activity as a CaCC, EGFR-kinase activity or the phosphorylation and dimerization status of EGFR and suggest that ANO1 and EGFR form a constitutive complex in HNSCC cell lines.

### Interaction of ANO1 and EGFR requires the trans-/juxta-membrane domain of EGFR

**Figure 2:**
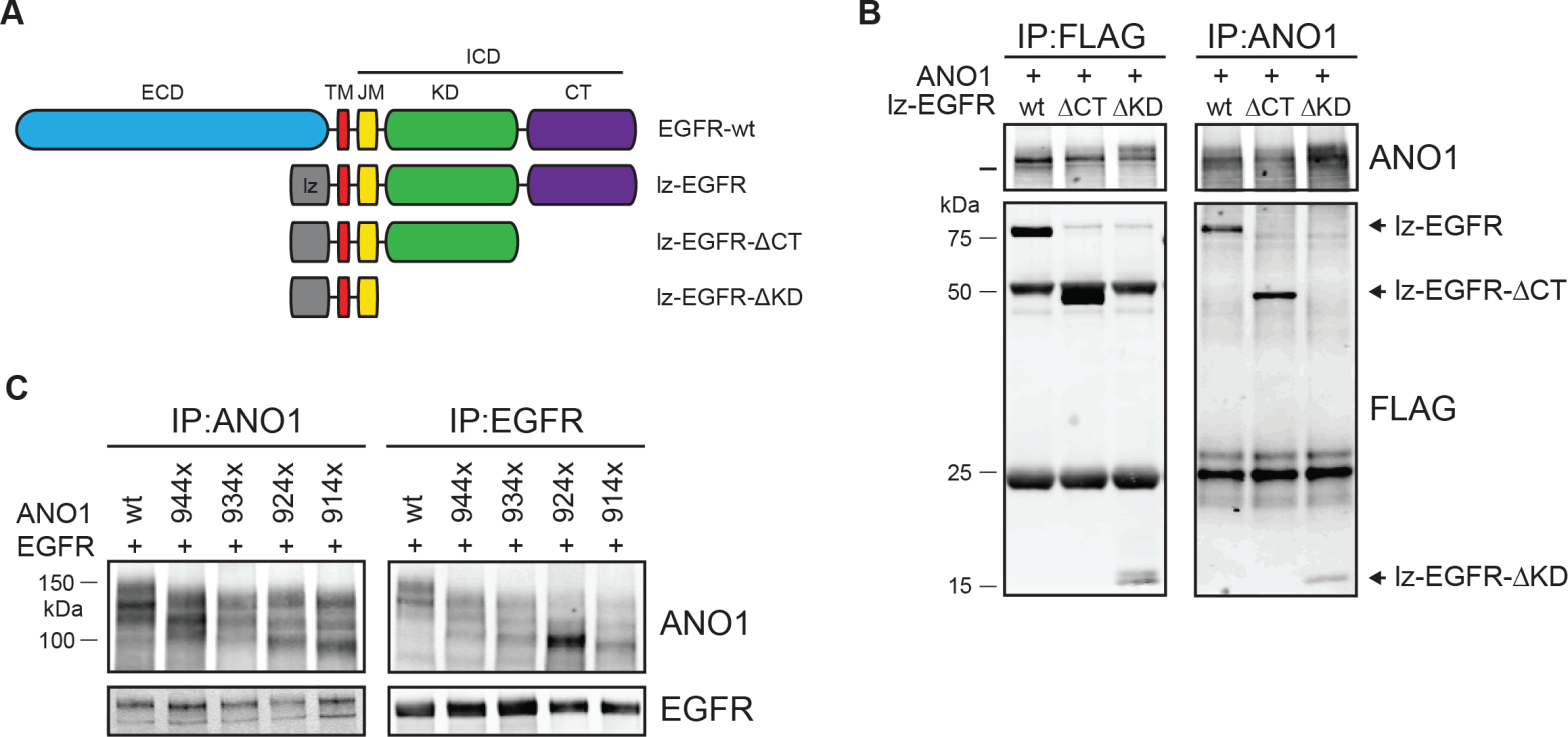
Interaction between ANO1 and EGFR involves the trans/juxtamembrane domain of EGFR **(A)** Schematic of the EGFR-constructs tested for interaction with ANO1. **(B)** Immunoprecipitation of ANO1 and FLAG-tagged truncation variants of lz-EGFR in HEK293T cell lysates. HEK293T cells were transfected with equal amounts of plasmids encoding ANO1 and lz-EGFR-variants. EGFR/ANO1 complexes were analyzed by immunoprecipitation using an anti-ANO1 or anti-FLAG antibody coupled to magnetic beads and immunoblotting of the eluted proteins. Representative immunoblots are shown. **(C)** Immunoprecipitation of lz-EGFR and ANO1 truncation variants in HEK293T cell lysates. HEK293T cells were transfected with equal amounts of plasmids encoding lz-EGFR and ANO1-variants and ANO1/lz-EGFR complexes were analyzed as in Figure 2B. The multiple bands for ANO1 represent different glycosylation variants of ANO1 [39].

The finding that lz-EGFR interacted with ANO1 to similar levels as wild-type EGFR (Figure 1I) demonstrates that the extracellular domain of EGFR is not required to form a complex with ANO1. To further investigate the structural requirements for the interaction between ANO1 and EGFR, we constructed deletion mutations of lz-EGFR lacking the C-terminal part of the protein or the C-terminal domain plus the kinase domain of EGFR (Figure 2A). Deletion of the complete intracellular domain of EGFR resulted in low expression levels and mislocalization of the protein to intracellular domains and was not tested for the interaction with ANO1 (data not shown). Coimmunoprecipitation using an ANO1- or FLAG-specific antibody revealed ANO1 to bind to all tested EGFR-constructs with similar affinity (Figure 2B). These results suggest that ANO1 interacts with the transmembrane domain and/or juxtamembrane domain of EGFR. To investigate the structural requirements of ANO1 for the interaction with EGFR we constructed deletion mutants of ANO1 lacking the 40–70 most C-terminal amino acids of ANO1. Expression on the plasma membrane was confirmed for all tested mutants (data not shown). Coimmunoprecipitation of ANO1 and EGFR showed that EGFR interacted with all mutants of ANO1 as determined by coimmunoprecipitation using an ANO1- or EGFR-specific antibody, suggesting that the C-terminus of ANO1 is not required for the interaction with EGFR (Figure 2C).

### EGFR-signaling increases ANO1-protein levels

**Figure 3:**
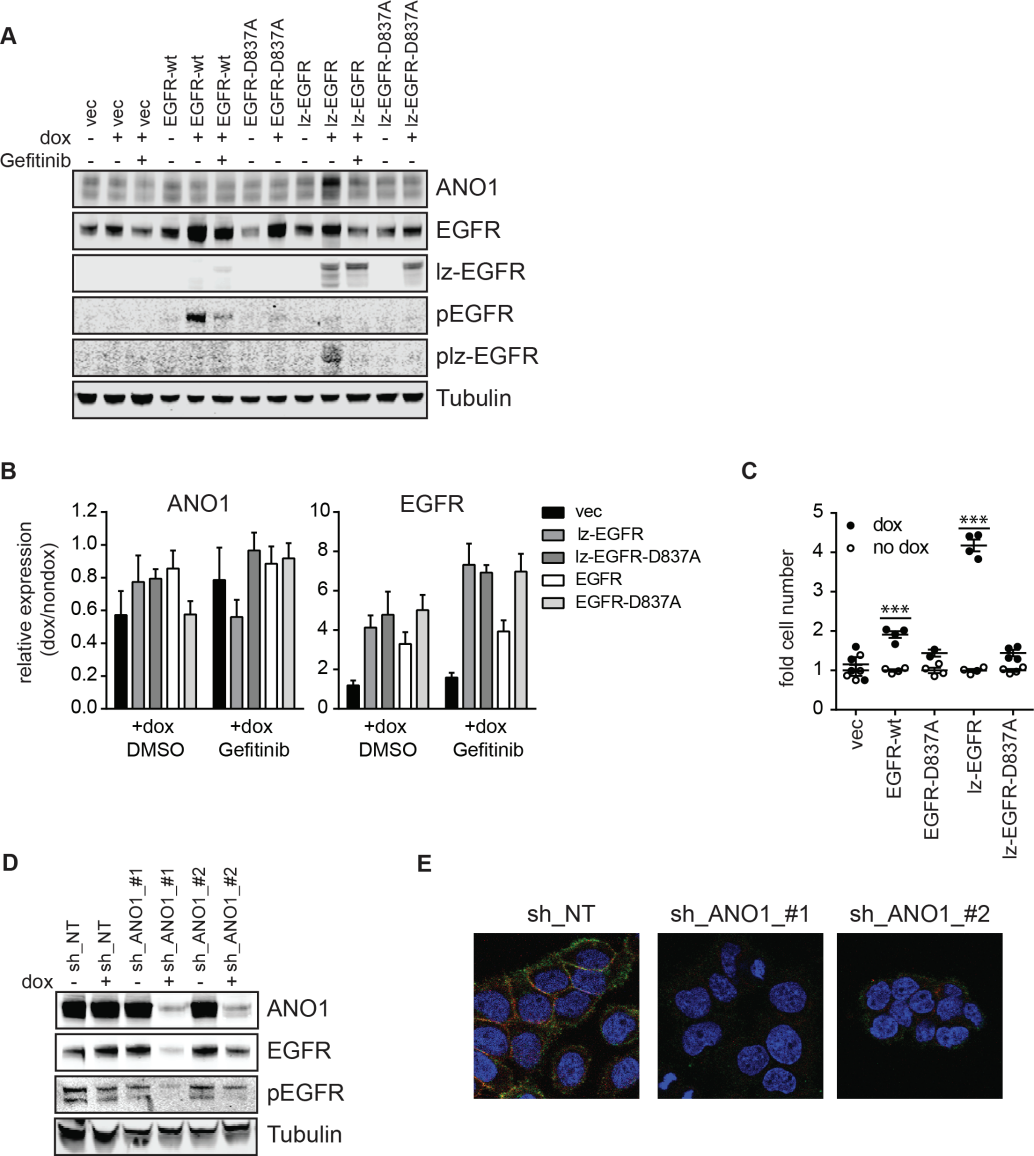
EGFR and ANO1 regulate each other's protein levels **(A)** Immunoblot of EGFR, phospho-EGFR and ANO1 protein levels in Te11 cells stably expressing dox-inducible expression constructs for EGFR-wt, -D837A, lz-EGFR or lz-EGFR-D837A or an empty vector control, in the presence or absence of dox (48 h) and Gefitinib (1 μM, 24 h). Tubulin served as a loading control. Representative immunoblots are shown. **(B)** Relative mRNA levels of EGFR and ANO1 in the same samples as used in A. mRNA-levels in dox-treated samples were normalized to the respective non-dox treated sample and are presented as the mean ± SEM of three independent experiments. **(C)** Relative cell proliferation of Te11 cells stably expressing the indicated dox-inducible constructs analyzed by Cell Titer Glo. Signals were normalized to the respective non-dox treated sample and are presented as the mean ± SEM of four independent experiments, *p* < 0.001*** as compared to respective no-dox condition. **(D)** Immunoblots of EGFR, phospho-EGFR and ANO1 protein levels in Te11 cells stably expressing dox-inducible shRNAs against ANO1 or a non-targeting control (NT) after treatment with dox for 72 h. Representative immunoblots are shown. **(E)** Immunofluorescence of ANO1 (green) and EGFR (red) in Te11 cells treated as in A analyzed by confocal microscopy. Representative images are shown.

Next, we set out to investigate potential functional consequences of the interaction between EGFR and ANO1. EGF has been shown to increase expression of ANO1 in a human bronchial epithelial cell line, indicating a positive feedback mechanism between EGFR-signaling and ANO1-expression. To test whether EGFR-signaling regulates ANO1-expression in cancer cells, we generated Te11 cells stably expressing a dox-inducible version of EGFR or lz-EGFR (Te11-EGFR, Te11-lz-EGFR). While activation of wildtype EGFR requires EGF, lz-EGFR has been shown to be constitutively phosphorylated and signaling active because of its constitutive dimerization [46]. Dox-induced expression of lz-EGFR in Te11 cells resulted in a significant increase of ANO1 protein levels, while expression of EGFR or a kinase-inactive mutant of lz-EGFR had no effect, suggesting that EGFR-signaling regulates ANO1 protein levels in cancer cells by an EGFR-kinase-activity-dependent mechanism (Figure 3A). Consistently, treatment with Gefitinib prevented the lz-EGFR induced increase in ANO1 protein levels and reduced ANO1-protein levels in the vector-expressing cells. The lz-EGFR induced increase in ANO1 protein levels led to a significant increase in calcium-dependent chloride current in Te11 cells, indicating that ANO1 is functional and localized on the membrane (Supplementary Figure 2). Notably, unlike previously reported for a bronchial epithelial cell lines, the increase of ANO1-protein levels in Te11 cells was not caused by an increase in ANO1-mRNA levels (Figure 3B), suggesting a posttranslational effect on ANO1-protein levels. To test whether the EGFR-signaling-induced increase in ANO1 protein levels had a functional effect on the proliferation rate of Te11 cells, we measured the viability of Te11-EGFR/lz-EGFR/-wt/-D837A cells in the presence and absence of dox (Figure 3C). Induction of EGFR- and lz-EGFR-expression resulted in a profound increase in cell proliferation, whereas the expression of the kinase-dead mutants had no effect. These results are consistent with a functional link between ANO1 and EGFR and support the hypothesis that EGFR regulate proliferation of cancer cells, in part, by increasing expression of ANO1.

### Knockdown of ANO1 reduces EGFR-protein levels

Knockdown of ANO1 inhibits EGFR-signaling in cancer cells, by a yet undefined mechanism. Having shown that ANO1 and EGFR form a functional complex and that EGFR-signaling regulates ANO1-protein levels in cancer cells, we wondered whether ANO1 would affect EGFR protein levels in these cells. As previously shown, treatment of Te11 cells expressing doxycycline (dox)-inducible shRNAs against ANO1 with dox resulted in a significant reduction of ANO1 protein levels and decrease of phosphorylated EGFR [33]. In addition to reducing the level of phosphorylated EGFR, we found that knockdown of ANO1 in Te11 cells using two independent shRNAs also led to a reproducible decline of EGFR protein levels that correlated with the efficiency of ANO1 knockdown (Figure 3D). Similarly, knockdown of ANO1 in Te11 cells markedly diminished the signal for EGFR as detected by immunofluorescence (Figure 3E). These findings suggest that expression of ANO1 directly regulates EGFR protein levels in cancer cells. To investigate the mechanism by which ANO1 affects EGFR protein levels and to test whether it involved regulation at the transcriptional level, we analyzed EGFR-mRNA levels after knockdown of ANO1 in Te11 cells. There was no consistent effect on the mRNA-level of EGFR after knockdown of ANO1 using two different shRNAs against ANO1 (Supplementary Figure 3A). These data suggest a post-transcriptional regulation of EGFR protein levels after ANO1 knockdown. The level of EGFR on the plasma membrane is tightly controlled by recycling/trafficking and degradation processes. Activation of EGFR triggers the endocytosis of the receptor and its rapid transport to the early endosomes from where it can be recycled back to the plasma membrane or sorted to lysosomes for degradation [47]. Thus, endosomal recycling and degradation are important regulators for EGFR protein level in cells. To test whether knockdown of ANO1 had an effect on the rate of EGF-induced EGFR-degradation, we stimulated Te11 cells expressing dox-inducible shRNAs against ANO1 (shRNA-ANO1-#1/#2) with EGF in the presence of dox and determined the amount of EGFR in the cells by immunoblotting (Supplementary Figure 3B). Stimulation of Te11 cells expressing a non-targeting control shRNA (NT) with EGF led to a time-dependent decrease in EGFR-protein levels, demonstrating the rapid rate of EGFR-degradation after EGF stimulation. Knockdown of ANO1 reduced EGFR-protein levels in all conditions, but did not affect the rate of EGF-induced degradation of EGFR (Supplementary Figure 3B). Having shown that ANO1 did not affect the rate of EGF-induced degradation of EGFR, we wondered whether ANO1 regulated EGFR-protein levels by affecting the steady-state degradation of the protein. In addition to lysosomal degradation, EGFR can be degraded via the proteasomal pathway [47]. For this, we treated Te11 cells with the proteasome inhibitor MG132 or Chloroquine, an inhibitor of lysosomal degradation and measured EGFR protein levels by immunoblotting after dox-induced knockdown of ANO1 (Supplementary Figure 3C). Neither treatment with MG132 nor with Chloroquine showed an effect on the ANO1-knockdown induced reduction of EGFR-protein levels, indicating that ANO1 does not affect the general turnover of EGFR in cancer cells. EGFR has been reported to be a target for protease-dependent degradation during the initiation of apoptotic pathways [48]. To exclude the possibility of a nonspecific reduction of EGFR-protein levels caused by a general increase in protein degradation due to apoptotic processes, we treated Te11 cells with inhibitors of calpain-proteases and a caspase-3 inhibiting peptide and measured EGFR protein levels after knockdown of ANO1 (Supplementary Figure 3D). Inhibition of neither protease was sufficient to prevent the reduction of EGFR-protein levels after knockdown of ANO1. Taken together, these results demonstrate that ANO1 regulates EGFR-protein levels in cancer cells by a posttranslational, degradation-independent mechanism, suggesting a role of ANO1 in stabilizing EGFR in the cells.

### Expression of EGFR rescues ANO1 protein levels and cell proliferation after knockdown of ANO1

**Figure 4:**
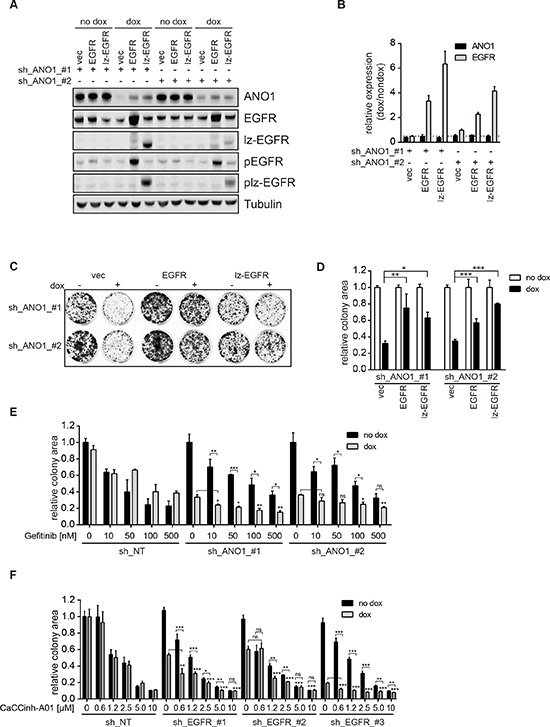
EGFR and ANO1 form a functional complex which regulates cancer cell proliferation **(A)** Immunoblots of EGFR, phospho-EGFR (Y1068) and ANO1 protein levels in Te11 cells stably co-expressing dox-inducible shRNAs against ANO1 and dox-inducible expression constructs for EGFR-wt, lz-EGFR or an empty vector control after treatment with dox for 72 h. Representative immunoblots are shown. **(B)** Relative mRNA-levels of ANO1 and EGFR in Te11 cells treated as in A. mRNA-levels in dox-treated samples were normalized to the respective non-dox treated sample and are presented as the mean ± SEM of three independent experiments. **(C)** Colony formation assay of Te11 cells stably co-expressing dox-inducible shRNAs against ANO1 and dox-inducible expression constructs for EGFR-wt, lz-EGFR or an empty vector control. Representative images are shown. **(D)** Quantification of the relative colony area of Te11 cells treated as in C. Values were normalized to the respective non-dox treated sample and are presented as the mean ± SEM of three independent experiments. ( *p* < 0.05*; *p* < 0.01**; *p* < 0.001***) **(E)** Relative colony area of Te11 cells stably expressing dox-inducible shRNAs against ANO1 or a non-targeting control (NT) after treatment with dox and/or Gefitinib. Values were normalized to the respective non-dox treated sample and are presented as the mean ± SEM of four independent experiments. Statistical analyses were performed using the Student's *t*-test or ANOVA with Tukey's post test as appropriate (*p < 0.05; **p < 0.01; ***p < 0.001); ns. not significant). **(F)** Relative colony area of Te11 cells stably expressing dox-inducible shRNAs against EGFR or a non-targeting control (NT) after treatment with dox and/or CaCCinh-A01. Values were normalized to the respective non-dox treated sample and are presented as the mean ± SEM of six independent experiments. Statistical analysis was performed as in Figure 4E.

Knockdown of ANO1 inhibited cell proliferation and reduced EGFR protein levels, whereas EGFR-signaling induced ANO1 protein levels in Te11 cells. We speculated that the loss of EGFR after knockdown of ANO1 might be responsible for the inhibitory effect on cell proliferation and that overexpression of EGFR under these conditions might rescue cell viability by recovering both, EGFR-signaling and ANO1 protein levels. To test this hypothesis we infected Te11-ANO1-shRNA-#1/#2 cells with constructs coding for dox-inducible versions of EGFR or lz-EGFR or an empty vector control. Dox-treatment in the resulting cell pool is expected to induce the expression of both, the shRNAs against ANO1 and the expression constructs for EGFR/lz-EGFR or empty vector, respectively. The technical feasibility of this system was tested by immunoblotting after treatment of the cells with dox or a solvent control (Figure 4A). Addition of dox resulted in a decrease of ANO1 protein levels in empty-vector-expressing cells for both shRNAs. Furthermore, dox induced a profound expression of EGFR-wt/lz in cells infected with the constructs coding for EGFR or lz-EGFR. The induction of EGFR-/lz-EGFR-expression was accompanied by a partial rescue of ANO1-protein levels in the cells (Figure 4A). The increase in ANO1-protein levels was not caused by an increase in ANO1-mRNA-levels as measured by quantitative PCR (Figure 4B), consistent with the results obtained by overexpression of EGFR in the absence of ANO1-shRNA. To test whether the EGFR-expression induced elevation of ANO1-protein levels was sufficient to rescue ANO1-knockdown-mediated inhibition of cell viability we measured cell viability using a colony formation assay (Figure 4C/D). Knockdown of ANO1 with both shRNAs significantly reduced the number of cells in vector-expressing cells. Dox-induced expression of both EGFR and lz-EGFR was sufficient to partially rescue ANO1-knockdown induced cell killing in the presence of both shRNAs (Figure 4C/D), consistent with the partial rescue of ANO1-protein levels observed (Figure 4A). Taken together, these results demonstrate that loss of ANO1 inhibits cell proliferation by reducing EGFR-expression and that it can be partially rescued by restoring EGFR-expression in the cells which subsequently leads to a recovery of ANO1-protein and cell viability. The bidirectional interplay of EGFR and ANO1 highlights the importance of the functional complex formed between both proteins in regulating proliferation of cancer cells.

### EGFR and ANO1 form a functional complex which regulates cancer cell proliferation

The observed interaction and functional link of EGFR and ANO1 suggests that ANO1 and EGFR jointly regulate EGFR-dependent pathways and cell proliferation in HNSCC and thus posts the question whether the efficacy of EGFR-targeted therapy can be increased by concurrent inhibition of EGFR and ANO1. To test this hypothesis we generated Te11 cells stably expressing doxycycline (dox)-inducible shRNAs against ANO1 or EGFR or a non-targeting control. Dox-induced knockdown of shRNAs against either EGFR or ANO1 alone led to inhibition of cell proliferation (Supplementary Figure 4 and [33]), indicating that both, EGFR and ANO1 are necessary and sufficient for proliferation in Te11 cells. To analyze the effect of simultaneous inhibition of EGFR and ANO1, we treated cells expressing ANO1-shRNAs with Gefitinib in the presence and absence of dox and measured cell proliferation (Figure 4E). Gefitinib inhibited cell proliferation in a concentration-dependent manner in all cell lines. Knockdown of ANO1 significantly decreased cell viability. Combination of ANO1 knockdown with Gefitinib treatment further reduced cell viability and colony formation. This effect was more pronounced with low concentrations of Gefitinib, indicating that inhibition of EGFR alone is sufficient to inhibit cell proliferation and that cotreatment of ANO1 is beneficial to improve inhibition of cell proliferation in the case of incomplete EGFR inhibition. Similarly, knockdown of EGFR in the presence of submaximal concentrations of the ANO1-inhibitor CaCCinh-A01 resulted in a significant additive inhibitory effect on cell proliferation (Figure 4F). Similar to the results observed with Gefitinib, the additive effect of simultaneous inhibition of ANO1 and EGFR was less pronounced with higher concentration of CaCCinh-A01, likely due to combinatorial off-target effects. This observation further supports the hypothesis that complete inhibition of either EGFR or ANO1 alone is sufficient to inhibit cell proliferation and that a combination of EGFR- and ANO1-inhibition can improve the effect of incomplete knockdown or enzyme inhibition by either inhibitor, which might delay or prevent the development of resistance to single agent treatment. These results suggest that EGFR and ANO1 jointly regulate cell proliferation by functioning in the same signaling node and are consistent with the hypothesis that co-targeting of ANO1 and EGFR could enhance the clinical potential of EGFR-targeted therapy in HNSCC.

### Expression of ANO1 predicts susceptibility to EGFR kinase inhibitors in HNSCC cell lines

**Figure 5:**
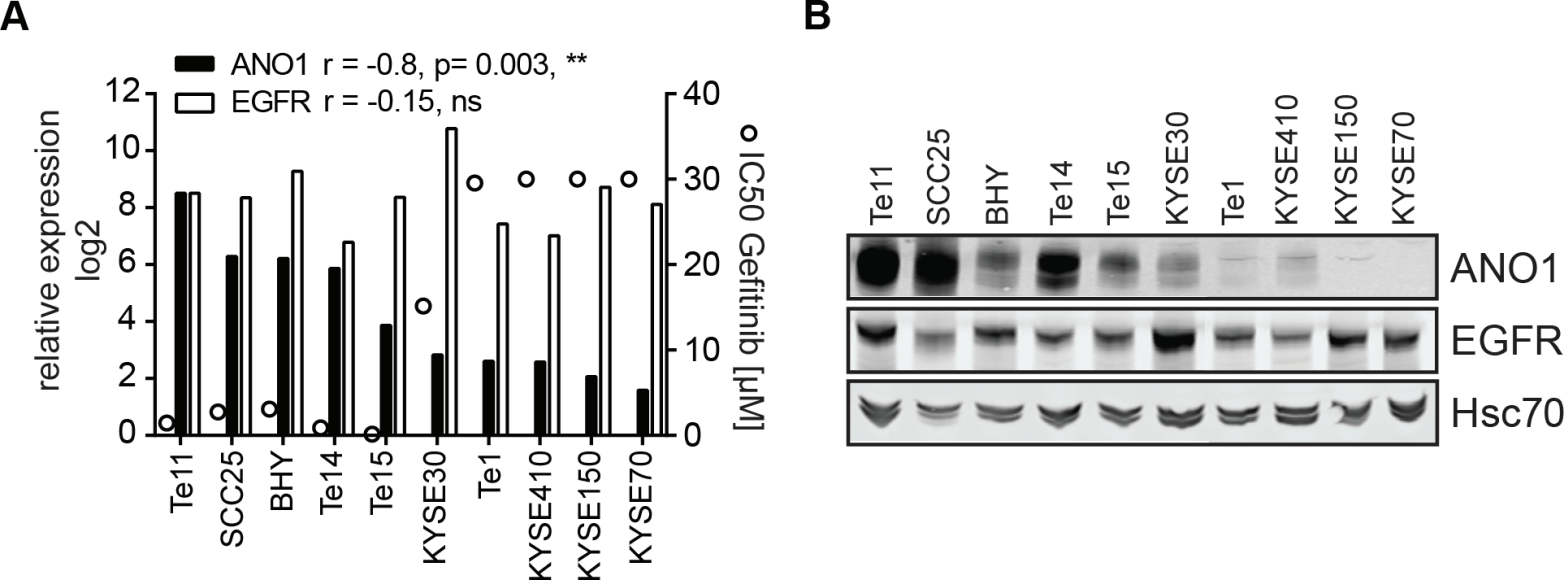
Expression of ANO1 predicts susceptibility to Gefitinib in HNSCC cell lines **(A)** Relative mRNA-levels of ANO1 and EGFR (bars, left y-axis) and sensitivity to Gefitinib (IC50, circles, right y-axis) of HNSCC cell lines, determined by quantitative PCR and Cell Titer Glo, respectively. A Pearson-correlation test was used to test for correlation between ANO1/EGFR expression and sensitivity to Gefitinib. **(B)** ANO1 protein levels in the HNSCC cell lines shown in Figure 5A.

HNSCC cell lines show differential sensitivity to EGFR-kinase inhibitors. EGFR expression alone is not sufficient to predict the response to EGFR-kinase inhibitors and activating EGFR kinase mutations are extremely rare in HNSCC [3], thus making the underlying mechanism for the differential sensitivity of HNSCC cells to EGFR inhibitors yet to be elucidated. To explore a potential association of ANO1 expression in HNSCC with clinical susceptibility to EGFR inhibitors, we tested a panel of HNSCC cell lines for sensitivity to Gefitinib and measured expression of EGFR and ANO1 using quantitative PCR and western blotting (Figure 5A and 5B). The results revealed a significant correlation between the expression of ANO1 and the sensitivity to Gefitinib ( *p* = 0.003, **; *r* = −0.8). A similar correlation was found between ANO1 amplification and sensitivity to Gefitinib (Supplementary Figure 5). The five cell lines with the highest expression of ANO1 (Te11, SCC25, BHY, Te14 and Te15) showed the highest sensitivity to Gefitinib with an IC50 < 1 uM. In contrast, Te1, KYSE140, KYSE150 and KYSE70 showed low mRNA-levels of ANO1 and an IC50 > 30 uM for Gefitinib, while KYSE30 showed an IC50 around 15 uM and intermediate expression of ANO1. Similar results were obtained with other EGFR kinase inhibitors (Supplementary Table 2). Taken together, these data show that ANO1 expression levels can be used as a biomarker to predict the sensitivity to EGFR kinase inhibitors.

## DISCUSSION

Since ANO1 was identified as a calcium-activated chloride channel [16–18], the mechanisms regulating its activity have remained elusive. Dysfunction of ANO1 is implicated in several disease states including cystic fibrosis, asthma, gastroparesis, hypertension, rota-virus induced diarrhea and polycystic kidney disease [26–30]. Therapeutic targeting of ANO1 is being actively investigated [49], understanding the mechanisms regulating ANO1′s activity could facilitate those efforts and support the development of novel therapies. An additional layer of complexity has recently been introduced by studies reporting channel-independent functions of ANO1, e.g. in regulating proliferation in head and neck cancer cells [39, 40]. In one of the studies, ANO1 was identified to function as a switch between the proliferative and metastatic state of HNSCC cells by interacting with the cytoskeletal protein Radixin [40], suggesting that ANO1 interacts with other proteins to promote cellular signaling and proliferation. Thus, ANO1 could be a target for anti-cancer therapy.

Here we report the first unbiased analyses of the interactome of endogenously expressed ANO1 in a HNSCC cell line. In contrast to a previous study [50] describing the interactome of ectopically expressed ANO1 in HEK cells after crosslinking, we used coimmunoprecipitation in native cell lysates to identify proteins interacting with ANO1 in a HNSCC cell line. The 40 proteins identified to interact with ANO1 in our study included cytoskeletal and calcium-binding proteins, transporters and membrane proteins. One of these proteins, SLC3A2, a subunit of an amino acid transporter and solute carrier was also identified by Perez-Cornejo et al. [50]. However knockdown of SLC3A2 using siRNA did not affect proliferation of Te11 cells, suggesting that SLC3A2 is not critical for ANO1′s effect on promoting cell proliferation (data not shown). While our approach favored the detection of natural, high-affinity interactions between endogenously expressed ANO1 and other endogenous proteins, overexpression of ANO1 in combination with crosslinking might stabilize low-affinity or low-abundance interactions, which might not be found under physiological conditions. Another explanation for the low number of overlapping proteins between the two studies is the different cell lines used. While Te11 cells express endogenous high levels for ANO1 but low levels of Radixin (previously identified to interact with ANO1 [50]), HEK cells do not express ANO1 endogenously and only low levels of EGFR, but high levels of Radixin. Notably, both studies did not identify any known ion channels or Anoctamins in the curated ANO1 interactome, despite significant levels of ANO1-mediated currents in the cells, suggesting that ANO1 on its own is sufficient to form a channel [51]. Furthermore it is notable that Calmodulin was not found in the ANO1 interactome, indicating that Calmodulin is not associated with and does not serve an adaptor for calcium-binding to ANO1 in Te11 cells as proposed by [52, 53].

Our discovery proteomics approach identified EGFR to interact with ANO1 in Te11, OE21 and SCC4 cells, suggesting ANO1 and EGFR interact in HNSCC cell lines. We found that the interaction between ANO1 and EGFR is mediated by the membrane/juxtamembrane domain of EGFR. The juxtamembrane domain (JMD) is a flexible stretch of 37AA connecting the intracellular with the helical transmembrane domain of EGFR and plays an important role in regulating the activity of EGFR [54]. In the inactive state of the EGFR receptor the positively charged JMD has been postulated to bind to the negatively charged inner leaflet of the membrane, holding the EGFR kinase domain in an inactive state [55, 56]. Upon binding of the ligand to the extracellular domain of EGFR, the intracellular domain undergoes conformational changes, resulting in the dissociation of the JMD from the membrane and the stabilization of the active conformation of the kinases (asymmetric dimer), leading to the activation of the receptor [54]. Factors interfering with or facilitating the formation of the asymmetric kinase dimer have been found to inhibit or activate EGFR signaling in cells, respectively [46, 57]. We have previously shown that ANO1 promotes EGFR-signaling in HNSCC cells [33]. The finding that ANO1 interacts with the trans/juxtamembrane domain of EGFR proposes the model, that ANO1 averts the autoinhibitory state of EGFR by hindering the JMD to bind to the inner leaflet of the membrane, thereby facilitating the activation of EGFR. This model is consistent with our findings that the kinase activity or the dimerization status of EGFR does not affect the interaction with ANO1. Interestingly, the JMD contains a predicted binding site for Calmodulin [58]. ANO1 has been shown to be functionally coupled to the IP3 receptor (IP3R1) in neurons from dorsal root ganglia [59]. The interaction of ANO1 and EGFR could foster the localization of EGFR to membrane regions proximal to intracellular calcium stores, favoring the calcium-dependent binding of Calmodulin to the JMD and ligand-dependent activation of EGFR, in addition to activation by other calcium activated kinase, like protein kinases protein kinase C. This model is further supported by our findings that knockdown of ANO1 reduces the phosphorylation of calmodulin-dependent kinases (CAMK) in breast cancer [33]. Further studies are needed to validate these models.

Furthermore, our data demonstrates that chloride transport of ANO1 is not required for its interaction with EGFR. This is consistent with our previous finding, that inhibiting the calcium-activated chloride transport of ANO1 is not sufficient to diminish ANO1-dependent cell proliferation in HNSCC [39]. Rather we have shown that the small molecule ANO1-inhibitor CaCCinh-A01 decreases HNSCC cell viability by facilitating the ER-associated, proteasomal degradation of ANO1, thereby reducing ANO1 protein levels and hence EGFR-signaling in the cells [33, 39].

In addition to the effect of ANO1 on the activation status of EGFR, our data points to a role of ANO1 in stabilizing EGFR protein levels. Knockdown of ANO1 led to a profound decrease in EGFR protein levels, without affecting EGFR mRNA levels. Furthermore, knockdown of ANO1 had no effect on EGF- induced or protease-dependent degradation of EGFR, suggesting that degradation of EGFR is not caused by unspecific protein degradation as a result of cell death. We found ANO1 protein levels in Te11 cells to be regulated by EGFR-signaling, demonstrating a bidirectional stabilization of ANO1 and EGFR protein levels likely as a result of the interaction between ANO1 and EGFR. Our finding that the stabilization of ANO1 protein levels in Te11 cells required the kinase activity of EGFR, suggest that EGFR might phosphorylate ANO1 directly or another protein involved in stabilizing ANO1, thereby increasing the stability of ANO1 protein in Te11 cells. Several tyrosine-phosphorylation sites in ANO1 have been predicted, however further studies are needed to explore the mechanisms behind EGFR-dependent stabilization of ANO1 protein levels in the cells in more detail.

As a consequence of the functional interplay between EGFR and ANO1, we found ANO1 and EGFR to jointly regulate proliferation in Te11 cells. Co-inhibition of EGFR and ANO1 had an additive effect on head and neck cancer cell proliferation, suggesting that co-targeting of ANO1 and EGFR could enhance the clinical potential of EGFR-targeted therapy in HNSCC and might delay or prevent resistance to single agent treatment. The lack of specific and potent inhibitors for ANO1 hampers the validation of this model *in vivo*. However, our data provide multiple lines of evidence, that combinatorial inhibition of EGFR and ANO1 might be beneficial for the treatment of HNSCC. While response of HNSCC patients to EGFR targeted therapy is not correlated to expression or amplification of EGFR [3, 14], we found that HNSCC cell lines with high expression of ANO1 showed enhanced sensitivity to Gefitinib. Gefitinib has been shown to prevent binding of ATP to EGFR regardless of the activation state of the kinase [60], hence favoring a model in which ANO1 expression increases dependence on EGFR-signaling rather than changing the sensitivity of EGFR to inhibition by Gefitinib. This model is consistent with ANO1 being a positive regulator of EGFR-signaling in HNSCC cells, thereby rendering the cells more sensitive to EGFR inhibition. One possible explanation of the increased dependence on EGFR-signaling in cells with high ANO1 expression is presented by our observation that inhibition of EGFR-signaling in Te11 cells caused a decrease in ANO1 protein levels (Figure 3A). Loss of ANO1 protein in Te11 cells was shown to inhibit cell proliferation and to induce apoptosis by reducing EGFR-signaling [33]. Hence inhibition of EGFR-signaling does not only diminish activation of EGFR downstream signaling pathways, but also initiates a negative feedback mechanism by reducing ANO1 protein levels.

In summary, our results introduce ANO1 expression as a predictive biomarker for the response to EGFR-targeting therapy in HNSCC and suggest combination of anti-EGFR and anti-ANO1 directed therapies as a promising therapeutic strategy for the treatment of HNSCC.

## EXPERIMENTAL PROCEDURES

### Cell culture

All HNSCC cell lines were maintained in RPMI, HEK293T cells in DMEM, supplemented with 10% fetal bovine serum at 37°C, 5% CO_2_. Te1, Te11, Te14 and Te15 cells were purchased from the RIKEN cell bank, BICR6 cells from Sigma, KYSE cell lines from DSMZ (the German cell bank), and all other cell lines from ATCC.

### Compounds

The compounds were purchased from the following vendors: Gefitinib, Erlotinib, Lapatinib, AEE788, Lafatinib, Z-DEVD-FMK, PD150606, MDL28170 (Tocris), MG132, Chloroquine (SigmaAldrich), CaCC_inh_-A01 (Specs). Compounds were dissolved in DMSO to a final concentration of 10 mM. Cells were treated with the indicated concentrations of inhibitors or matching volumes of DMSO.

### Plasmids

Generation of plasmids coding for ANO1(abc) was described in [43], EGFR and lz-EGFR in [46]. Sequences for EGFR and lz-EGFR were cloned into the pLVX-tetone-puro vector (Clontech). Mutations and truncations were generated by site-directed mutagenesis (Q5 mutagenesis kit, New England Biolabs). The sequence identity for all plasmids was verified by Sanger sequencing (Genewiz). ANO1-shRNA and virus generation were described in [33]. The following shRNA sequences for EGFR were used:

shRNA_#1: CAATTCCACCGTGGCTTGCATCTCGAGATGCAAG CCACGGTGGAATTG

shRNA_#2: GCTGAGAATGTGGAATACCTACTCGAGTAGGTAT TCCACATTCTCAGC

shRNA_#3: AGAATGTGGAATACCTAAGGCTCGAGCCTTAGGT ATTCCACATTCTC

Copy number analysis and quantitative PCR were performed as described in [33].

### Cell viability and colony formation assays

For measurement of cell viability, 3 × 10^3^ cells per well were seeded into a 96-well plate, adhered overnight and treated with the indicated concentrations of inhibitor or solvent for 72 h. Cell viability was assessed Cell Titer Glo (Promega). Colony formation assays were performed by seeding 1000 cells/well cells in 24-well plates. Cells were allowed to adhere overnight before treatment with the indicated concentrations of inhibitor or DMSO. Colonies were stained after 10–18 days with 0.2% crystal violet in PBS/4% formalin. Colony area was quantified using the Odyssey scanner and software (LICOR).

### Immunoprecipitation

HNSCC cells were seeded on 15 cm dishes and allowed to grow to 70% confluence. 3 × 10^6^ HEK293T cells were seeded on 10 cm dishes, allowed to adhere overnight and were transfected with 3 ug DNA using 18 ul of FuGene 6 (Promega). Cells were lysed in RIPA buffer (Cell Signaling) and ~500 ug of total protein was incubated with an anti-EGFR (Cell Signaling, #2256), ANO1 (SP31, Abcam) and FLAG (M2, Sigma-Aldrich) antibody for 1 h at 4C. Antibodies were precipitated using ProteinG coated Dynabeads (Invitrogen) and bound protein was eluted in Laemmli buffer (Invitrogen) for 10 min at 70C.

### Western blotting

Cell lysates were analyzed by SDS-PAGE and western blotting using standard protocols and the following primary antibodies: anti-ANO1 (Abcam, SP31), anti-EGFR (Cell Signaling), anti-Tubulin (Sigma), anti-FLAG (Sigma-Aldrich). Blots were analyzed using an Odyssey scanner (LICOR).

### Protein identification

In-gel trypsin digestion of gel lanes with the immunoprecipitated proteins and subsequent identification by LC-MS was performed as described [61]. Database searches were done with Mascot (version 2.4, Matrix Science) against the UniProt database (release of April 2013). Protein identifications were validated and summarized in Scaffold (version 4.0.3, Proteome Software Inc.), setting protein identifications thresholds at 99% protein confidence and 2 unique peptides at 90% peptide confidence, corresponding to 0.0% protein FDR and 1.6% peptide FDR respectively. The resulting protein lists were further refined using two filters. First, the list was reduced to only those proteins that were identified in all three experiments. Because these will include common background proteins, in a second step the identified proteins were annotated with a ‘CRAPome score’ (C-score) according to the CRAPome repository, a published analysis of frequent hitters in pulldown experiments [62]. The C-score describes the number of experiments (of 334) in the CRAPome-database in which the protein is listed. Based on the frequency distribution of that database, proteins with a frequency above 0.1 were considered to be unspecific. The complete list of identified proteins is included as Supplementary Table 1.

Immunofluorescence was performed as described in [39].

YFP quench assay Te11-lzEGFR cells were pretreated with or without dox for 24 h, transiently transfected with pBM-YFPquench H148Q/I152L [43], FACS sorted for YFP and plated in 96 well plates. ANO1-dependent chloride flux in the presence of 150 uM UTP was assesses 72 h after transfection by analyzing quenching of YFP-fluorescence as described in [43].

### Statistical analysis

All data are expressed as means ± s.e.m. Statistical analyses were performed in GraphPadPrism using the Student's *t*-test or ANOVA with Tukey's post test as appropriate (*p < 0.05; **p < 0.01; ***p < 0.001); ns. not significant).

## SUPPLEMENTARY FIGURES AND TABLES




