# Supplementary material for: ANO1/TMEM16A interacts with EGFR and correlates with sensitivity to EGFR-targeting therapy in head and neck cancer
Source: Oncotarget. 2015 Mar 16;6(11):9173–88. doi: 10.18632/oncotarget.3277 (PMC4496210; doi:10.18632/oncotarget.3277)
Supplement: Supplementary file 1 [file oncotarget-06-9173-s001.pdf]

SUPPLEMENTARY FIGURES AND TABLES

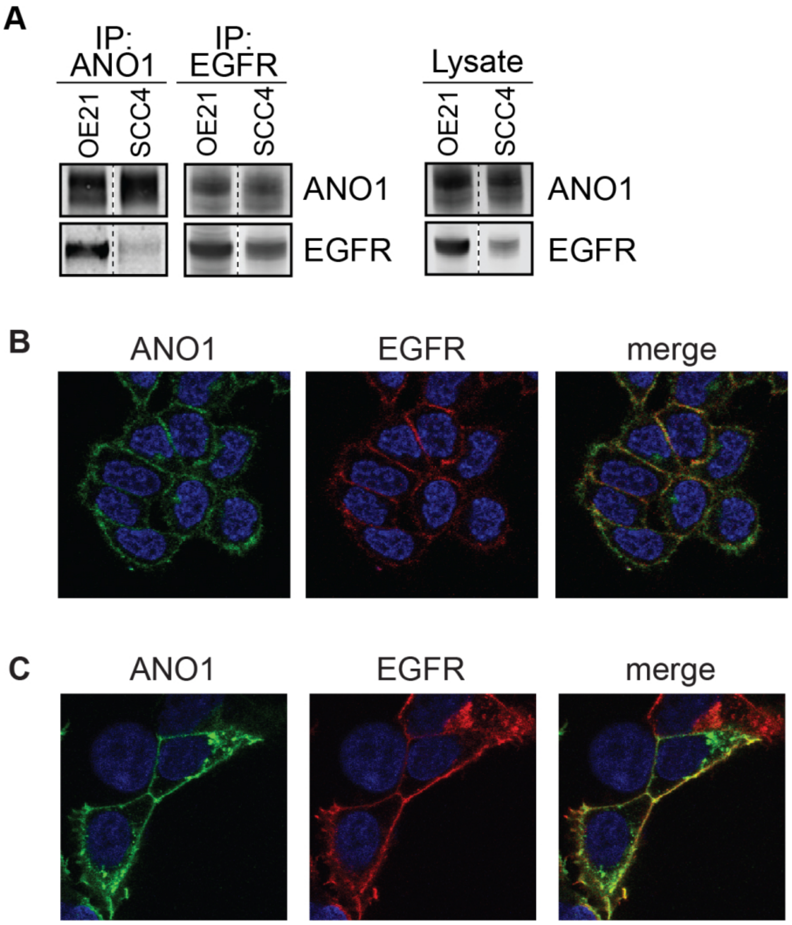

Supplementary Figure S1: ANO1 and EGFR interact. A. Immunoblot after immunoprecipitation of ANO1 (left) or EGFR (middle) from OE21 and SCC4 cell lysates (right) using an anti-ANO1 or anti-EGFR antibody coupled to magnetic beads. Eluted proteins were run on a western blot and probed with antibodies against ANO1 and EGFR. Representative immunoblots are shown. **B.** Immunofluorescence of ANO1 (green) and EGFR (red) in Te11 cells analyzed by confocal microscopy. Representative images are shown. **C.** Immunofluorescence of ANO1 (green) and EGFR (red) in HEK293T cells transfected with equal amounts of plasmids coding for ANO1 and EGFR analyzed by confocal microscopy. Representative images are shown.

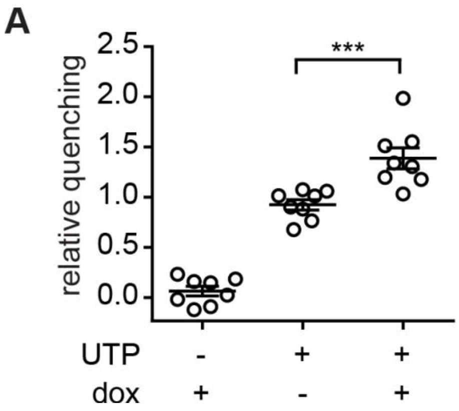

Supplementary Figure S2: Expression of lz-EGFR increases ANO1 protein levels and ANO1-dependent chloride currents in Te11 cells. A. Te11 lz-EGFR cells were pretreated with or without dox, transiently transfected with YFP-H148Q/I152L and ANO1-dependent chloride currents were assessed by measuring quenching of YFP-fluorescence. Data are presented as the average ± SEM of normalized values, $n = 8$, ***$p < 0.001$.

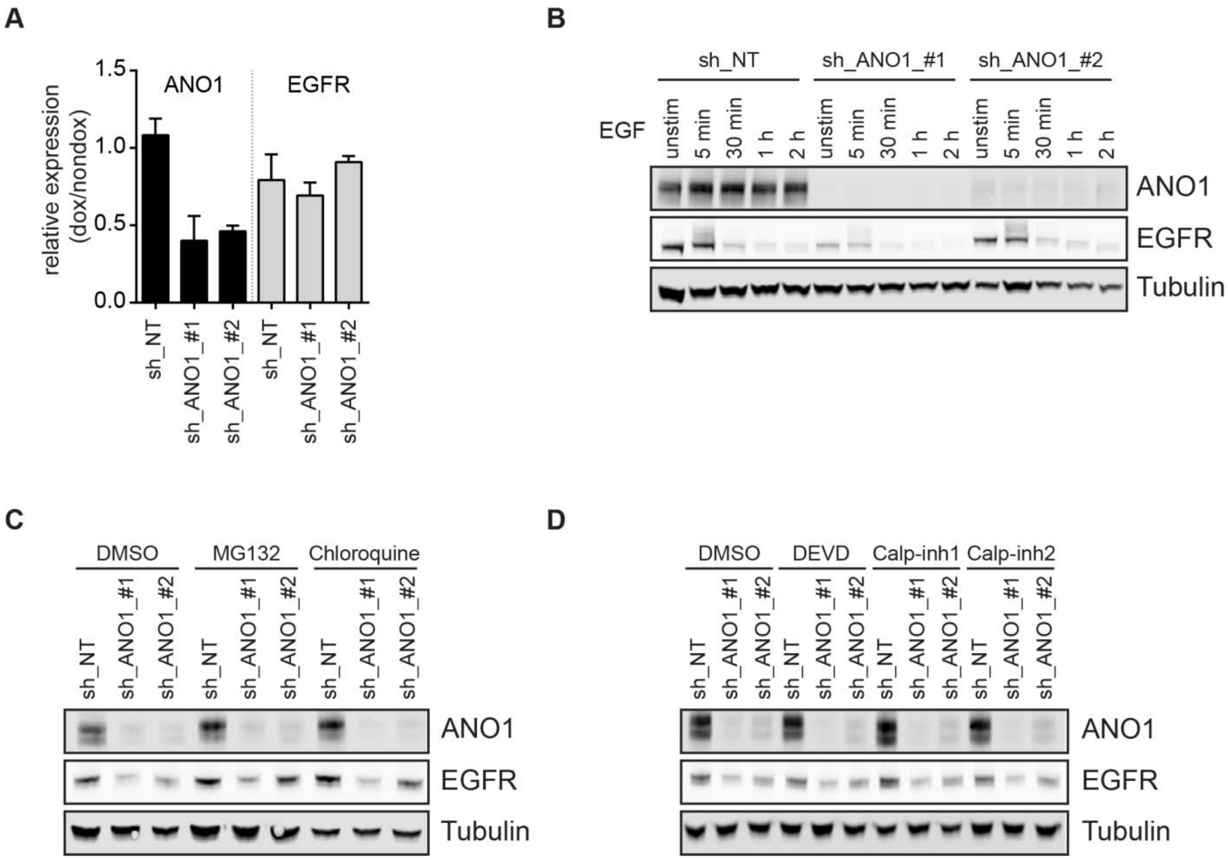

Supplementary Figure S3: Knockdown of ANO1 reduces EGFR protein levels. A. Relative mRNA-levels of ANO1 and EGFR in Te11 cells treated as in Figure 3D. mRNA-levels in dox-treated samples were normalized to the respective non-dox treated sample and are presented as the mean ± SEM of three independent experiments. **B.** Immunoblots of EGFR and ANO1 protein levels in Te11 cells after knockdown of ANO1 and stimulation with 100 ng/ml EGF for the indicated time periods. Representative immunoblots are shown. **C.** Immunoblots of EGFR and ANO1 protein levels in Te11 cells after knockdown of ANO1 and treatment with MG132 (15 µM), Chloroquine (100 µM) or DMSO. Representative immunoblots are shown. **D.** Immunoblots of EGFR and ANO1 protein levels in Te11 cells after knockdown of ANO1 and treatment with Z-DEVD-FMK, (100 µM), Calp-inh1 (PD150606, 20 µM), Calp-inh2 (MDL28170, 20 µM) or DMSO. Representative immunoblots are shown.

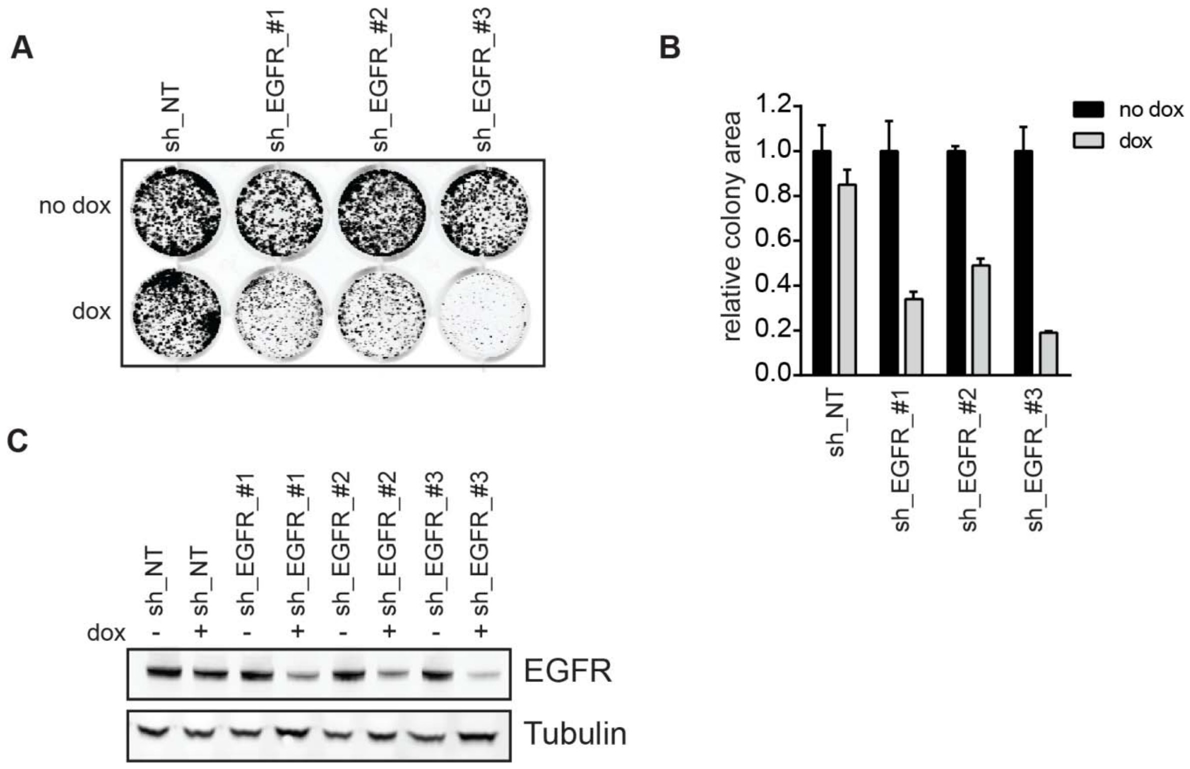

Supplementary Figure S4: Knockdown of EGFR reduces cell viability. A. Colony formation assay of Te11 cells stably expressing dox-inducible shRNAs against EGFR or a non-targeting control. Colonies were stained 10 days after addition of dox. **B.** Quantification of the relative colony area of Te11 cells treated as in A. Values were normalized to the respective non-dox treated sample and are presented as the mean ± SEM of three independent experiments. **C.** Immunoblot of EGFR protein levels in Te11 cells stably expressing dox-inducible shRNAs against EGFR or a non-targeting control after treatment with dox for 3 days.

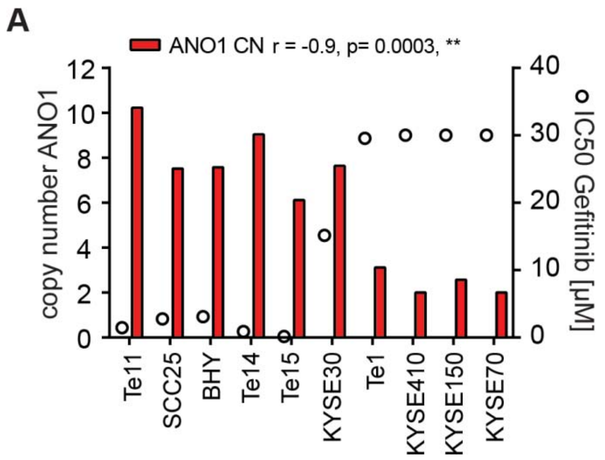

Supplementary Figure S5: Amplification of ANO1 correlates with sensitivity to Gefitinib. A. Copy number of ANO1 (bars, left y-axis) and sensitivity to Gefitinib (IC50, circles, right y-axis) of HNSCC cell lines, determined by genomic PCR and Cell Titer Glo, respectively. A Pearson-correlation test was used to test for correlation between ANO1 copy number and sensitivity to Gefitinib.

Supplementary Table S1. Proteins identified in all three experiments with 2 or more unique peptides

Supplementary Table S2. Expression of ANO1 correlates with sensitivity to EGFR-targeted therapy Table summarizes the effect of EGFR kinase inhibitors on the proliferation of head and neck cancer cell lines. IC50 values (µM) of the inhibition of cell proliferation (CellTiter glow assay) are given as the mean of at least three experiments.

	IC50 (µM)					
	Gefitinib	Erlotinib	Lapatinib	Afatinib	AEE788	
Te11	1.45	1.00	0.41	0.32	0.22	high ANO1 expression
SCC25	2.74	2.08	2.39	2.36	1.29	
BHY	3.08	1.50	2.58	0.21	1.40	
Te14	0.87	3.78	1.29	0.06	0.80	
Te15	0.15	0.02	0.23	0.07	0.14	
KYSE30	15.16	30.00	8.32	9.97	16.25	low ANO1 expression
Te1	29.55	20.47	15.98	5.19	10.64	
KYSE410	30.00	11.01	5.98	3.91	2.44	
KYSE150	30.00	30.00	6.95	30.00	7.96	
KYSE70	30.00	15.75	9.61	4.26	5.67	
